# Supplementary material for: TLR2 Supports γδ T cell IL-17A Response to ocular surface commensals by Metabolic Reprogramming
Source: bioRxiv. 2024 Apr 1:2024.04.01.587519. Preprint. [Version 1] doi: 10.1101/2024.04.01.587519 (PMC11071315; doi:10.1101/2024.04.01.587519)
Supplement: Supplementary file 1 [file media-1.pdf]

Supplemental Figures

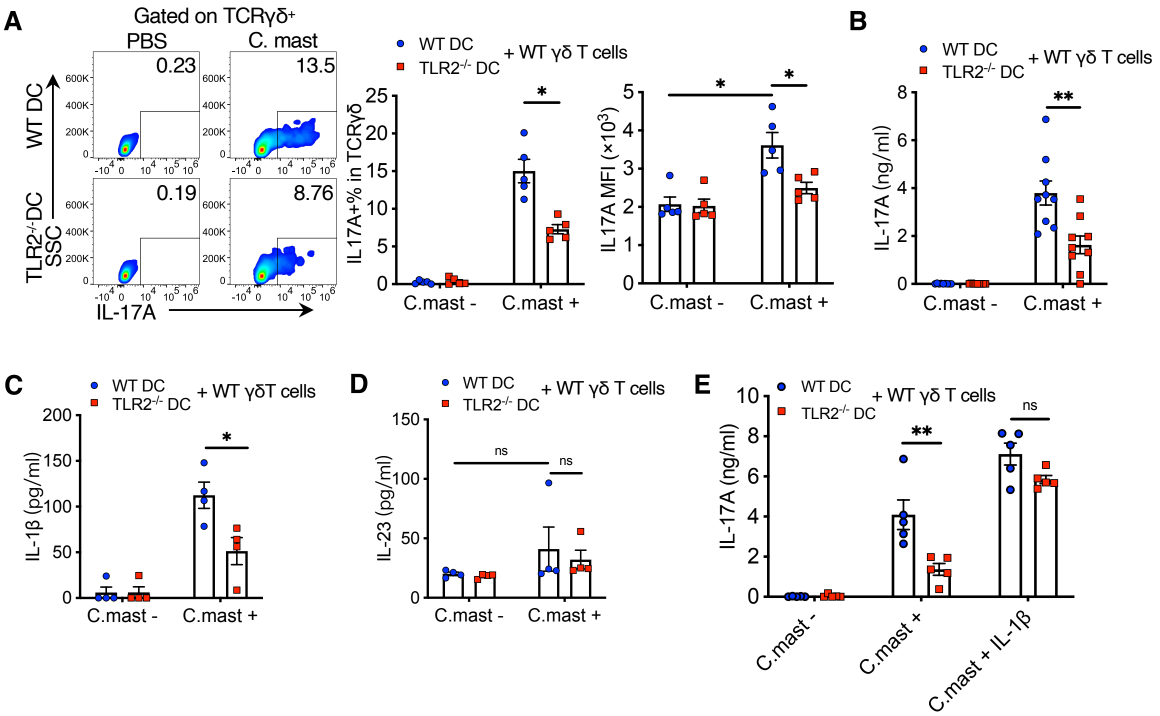

Figure S1 (refers to Figure 2). TLR2 expressed by DC drives IL-1β needed to support the γδ T cell IL-17A response to *C. mast*

(A-E) WT γδ T cells were isolated from WT mice and co-cocultured with WT or TLR2$^{-/-}$ CD11c+ dendritic cells for 72 hours (γδ T cells: 2×10^4; CD11c+ cells: 1×10^5) with/ without heat-killed *C. mast*. Supernatants and cells were collected for ELISA and flow cytometry experiments.
(A) Representative FACS plots and bar graphs showing the percentage and mean fluorescence index (MFI) of IL-17A in γδ T cells from WT or TLR2$^{-/-}$ DC co-culture. N=5. Combined data from 3 experiments.
(B-D) Bar graphs presenting the levels of IL-17A **(B)**, IL-1β **(C)**, and IL-23 **(D)** proteins in the supernatants from WT or TLR2$^{-/-}$ DC co-culture system. Data was combined from 4 experiments (N=9) for (**B**) and 2 experiments (N=4) for **(C-D)**.
(E) IL-1β (100ng/ml) was added to the co-culture system for 72h, and the IL-17A level was measured by ELISA. N=5. Combined data from 3 experiments.
Statistical significance was determined by Welch's t-test (right box of panels B-E) or the Mann-Whitney U test (right box of panel A). (A-E) Bars represent mean ± SEM with *P<0.05, **P<0.01, ***P <0.001.

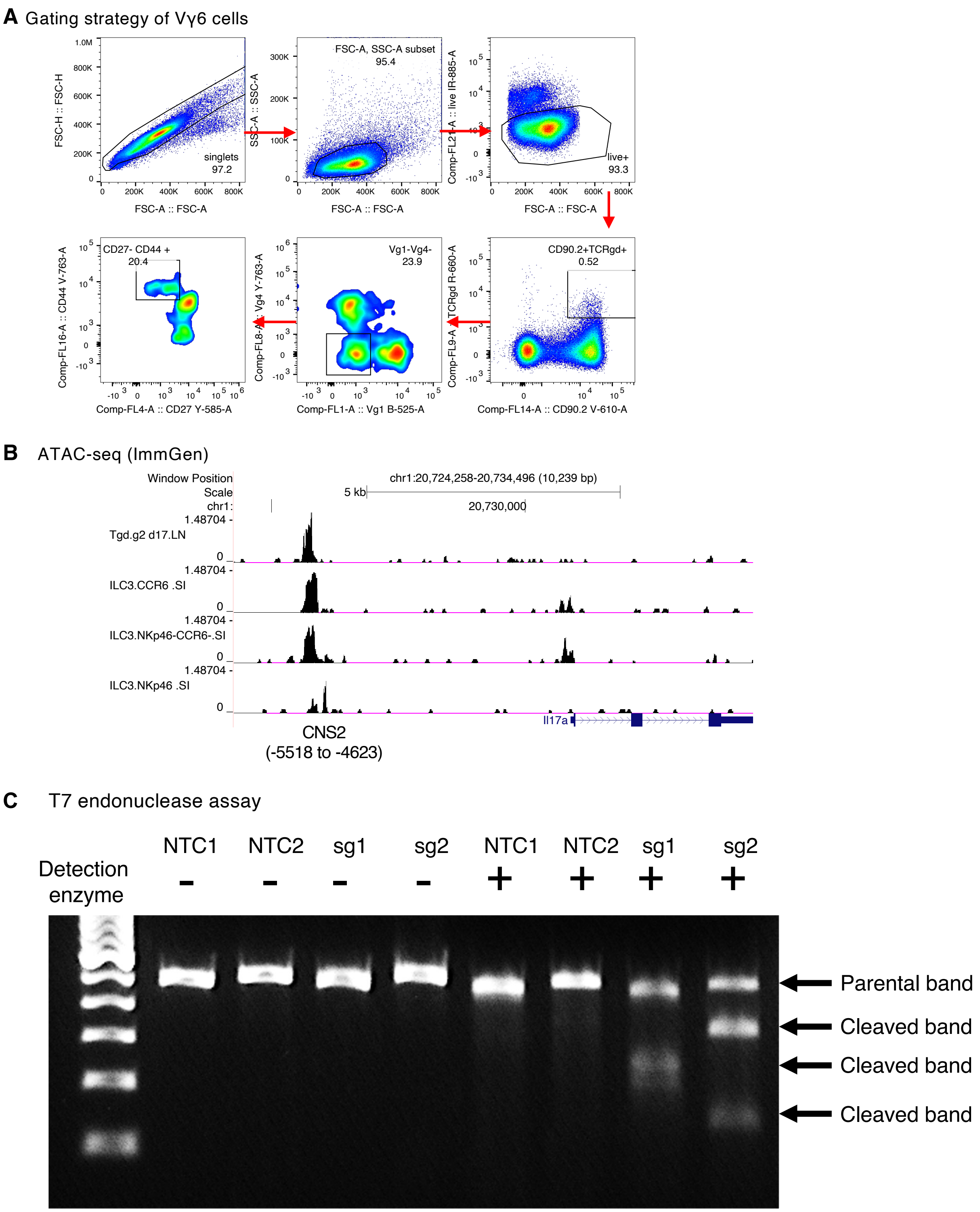

Figure S2 (refers to Figure 5). Decreased IκBζ is causally related to deficient TLR2-dependent IL-17A production in Vγ6 cells

(A) Representative FACS plots showing CD27^{-}CD44^{high} Vγ1-Vγ4- gating strategy from eye draining lymph nodes of *C. mast*$^{-}$ or *C. mast*$^{+}$ TLR2$^{-/-}$ mice for RNA-seq and ATAC-seq.
(B) The UCSC genome browser-depicted traces, to the same scale, for the Il17a genomic region of the indicated cell subsets using ATAC-seq under the ImmGen Program.
(C) Vγ6 cells were transfected with CRISPR ribonucleoprotein complexes targeting different regions of Nfkbiz (sg1 and sg2) or a non-targeting control(NTC) that does not target the mouse reference genomes. PCR products specific to the Nfkbiz target region were analyzed by the T7 E1 mismatch cleavage assay. Cleaved bands indicate successful CRISPR editing.

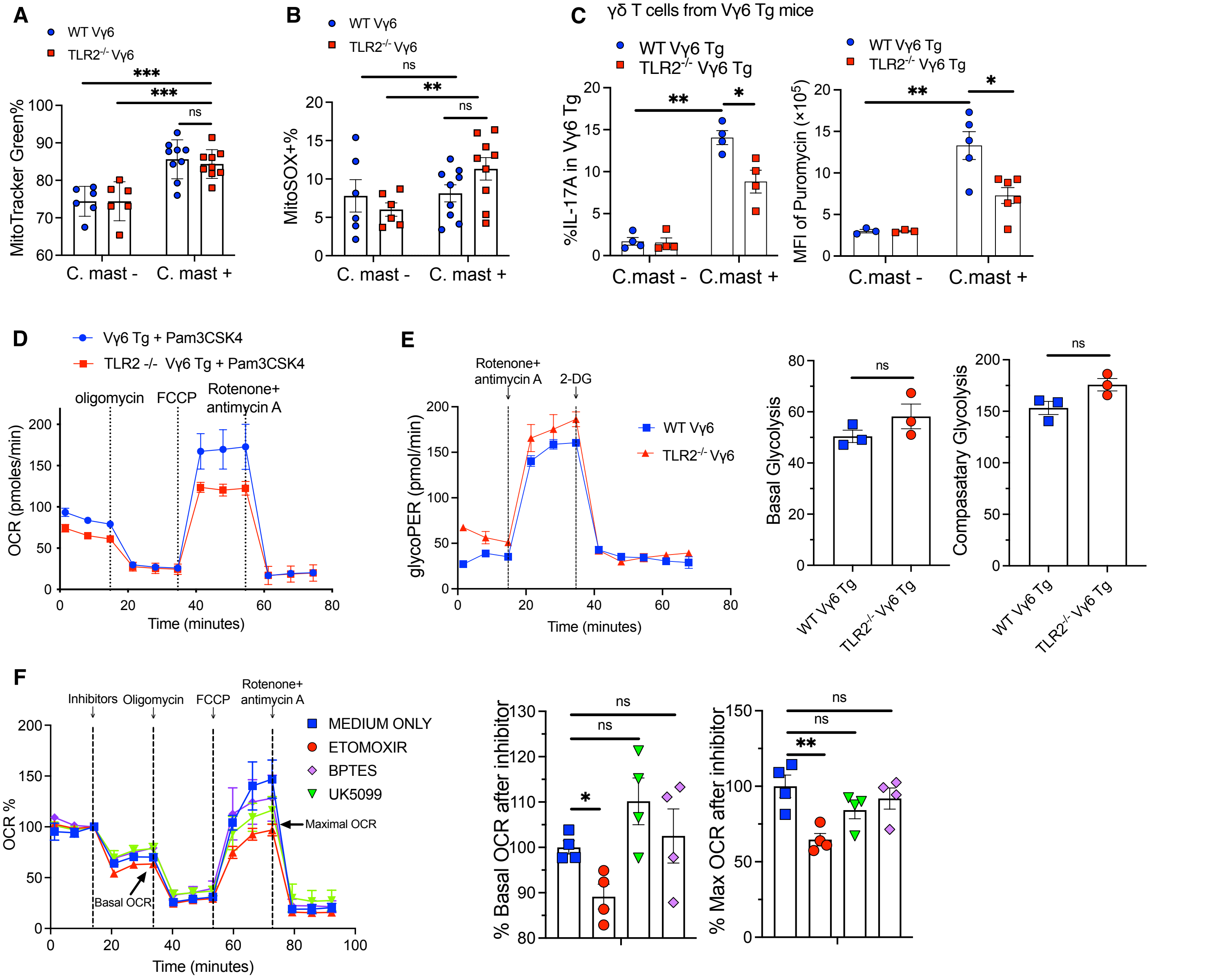

Figure S3 (refers to Figure 6). TLR2 deficiency Vγ6 T cells do not impair glycolysis

γδ T cells were collected from secondary lymphoid tissues of TLR2 -/- or WT mice and subjected to Seahorse Metabolic profiling.
(A-B) FACS plots and bar graphs showing the percentage of MitoTrack Green **(A)** and MitoSOX (mitochondrial Superoxide Indicator **(B)** in Vγ6 cells from WT and $TLR2^{-/-}$ mice. N= 3–6. Combined data from 2 independent experiments.
(C) Vγ6 cells isolated from WT and $TLR2^{-/-}$ Vγ6 Tg mice were co-cocultured with WT $CD11c^{+}$ dendritic cells for 72 hours (γδ T cells: 2×10^{4}; CD11c+ cells: 1×10^{5}) with or without heat-killed *C. mast*. The percentage of $IL\text{-}17A^{+}$ cells (left plot) was measured by flow cytometry after staining for anti-IL17A. For ATP measurement, puromycin was added to the culture for the last 1 hour and then Vγ6 cells were stained with anti-puromycin. The MFI of incorporated puromycin reflects ATP production. N= 3–6.
(D) Vγ6 cells were sorted from WT and $TLR2^{-/-}$ Vγ6 Tg mice treated with Pam3CSK4 for 3 days and then were subjected to the Seahorse Mito stress test. Representative data from one of 3 independent experiments.
(E) Vγ6 T cells isolated from WT and $TLR2^{-/}$ *C. mast*+ Vγ6 transgenic mice were subjected to GlycoPER assay. Bar graphs show basal Glycolysis and compensatory glycolysis of WT and $TLR2^{-/-}$ Vγ6 cells. N= 3. Representative data from one of 2 independent experiments.
(F) Vγ6 cells in *C. mast*$^{+}$ Vγ6 transgenic mice were sorted and performed seahorse substrate oxidation stress test. Inhibitors (ETOMOXIR, UK5099, and BPTES) were injected before oligomycin treatment. Percentage of basal OCR after inhibitors injection and the percentage of maximal OCR after inhibitor injection were calculated and displayed in bar plots. N= 4. Representative data from one of 2 independent experiments.
Significance was determined by the Whitney U test in (A, C-E) or Welch's t-test (F).

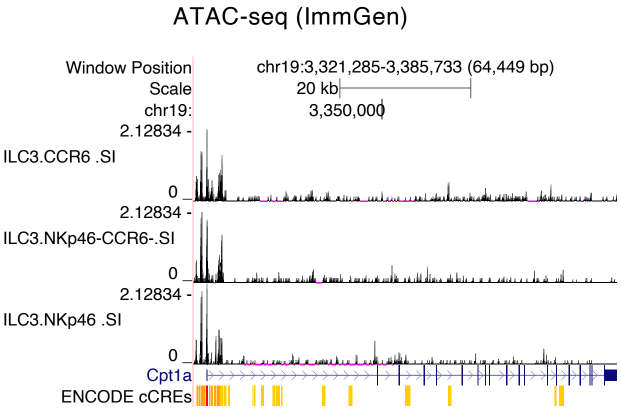

Figure S4 (refers to Figure 7)

UCSC genome browser output depicting ATAC-seq traces for the promoter and enhancer regions (ENCODE cCREs) of *Cpt1a* in indicated IL-17A producing immune cells sequenced by ImmGen program.
